# Supplementary material for: Light and electron microscopic features of preclinical pseudoexfoliation syndrome
Source: PLoS One. 2023 Mar 9;18(3):e0282784. doi: 10.1371/journal.pone.0282784 (PMC9997979; doi:10.1371/journal.pone.0282784)
Supplement: Attachment — Submitted filename: LM EM Point-by-point response .docx [file pone.0282784.s001.docx]

Dear Prof. Sanjoy Bhattacharya,

The authors would like to thank you and the reviewers for the support and thoughtful critique of our manuscript. Please find attached a revised version of the manuscript (PONE-D-22-32461) entitled “Light and Electron Microscopic Features of Preclinical Pseudoexfoliation Syndrome”. Our responses to the reviewers’ comments are shown in red and enclosed below. We hope that these changes strengthen the manuscript so that it may be accepted for eventual publication in PLOS ONE. We look forward to hearing from you and the reviewers, and please feel free to contact us with any comments or questions.

Best regards,

Yanin Suwan, MD

Department of Ophthalmology

Ramathibodi Hospital, Mahidol University

Rama VI, Bangkok

Thailand, 10400.

Email: [yanin.suwan@gmail.com](mailto:yanin.suwan@gmail.com)

Journal requirements:

When submitting your revision, we need you to address these additional requirements.

1.  Please ensure that your manuscript meets PLOS ONE's style requirements, including those for file naming. The PLOS ONE style templates can be found at

<https://journals.plos.org/plosone/s/file?id=wjVg/PLOSOne_formatting_sample_main_body.pdf>  and

<https://journals.plos.org/plosone/s/file?id=ba62/PLOSOne_formatting_sample_title_authors_affiliations.pdf>

2. We note that you have stated that you will provide repository information for your data at acceptance. Should your manuscript be accepted for publication, we will hold it until you provide the relevant accession numbers or DOIs necessary to access your data. If you wish to make changes to your Data Availability statement, please describe these changes in your cover letter and we will update your Data Availability statement to reflect the information you provide.

3. Please review your reference list to ensure that it is complete and correct. If you have cited papers that have been retracted, please include the rationale for doing so in the manuscript text, or remove these references and replace them with relevant current references. Any changes to the reference list should be mentioned in the rebuttal letter that accompanies your revised manuscript. If you need to cite a retracted article, indicate the article’s retracted status in the References list and also include a citation and full reference for the retraction notice.

Additional Editor Comments:

All three learned reviewers have requested minor revision that can be done by incorporating appropriate changes in the manuscript


[Note: HTML markup is below. Please do not edit.]

Reviewers' comments:

Reviewer's Responses to Questions

**Comments to the Author**

1. Is the manuscript technically sound, and do the data support the conclusions?

The manuscript must describe a technically sound piece of scientific research with data that supports the conclusions. Experiments must have been conducted rigorously, with appropriate controls, replication, and sample sizes. The conclusions must be drawn appropriately based on the data presented.

Reviewer #1: Yes

Reviewer #2: Yes

Reviewer #3: Yes

2. Has the statistical analysis been performed appropriately and rigorously?

Reviewer #1: Yes

Reviewer #2: Yes

Reviewer #3: Yes

3. Have the authors made all data underlying the findings in their manuscript fully available?

The [PLOS Data policy](http://www.plosone.org/static/policies.action#sharing) requires authors to make all data underlying the findings described in their manuscript fully available without restriction, with rare exception (please refer to the Data Availability Statement in the manuscript PDF file). The data should be provided as part of the manuscript or its supporting information, or deposited to a public repository. For example, in addition to summary statistics, the data points behind means, medians and variance measures should be available. If there are restrictions on publicly sharing data—e.g. participant privacy or use of data from a third party—those must be specified.

Reviewer #1: Yes

Reviewer #2: Yes

Reviewer #3: Yes

4. Is the manuscript presented in an intelligible fashion and written in standard English?

PLOS ONE does not copyedit accepted manuscripts, so the language in submitted articles must be clear, correct, and unambiguous. Any typographical or grammatical errors should be corrected at revision, so please note any specific errors here.

Reviewer #1: Yes

Reviewer #2: Yes

Reviewer #3: Yes

5. Review Comments to the Author

Please use the space provided to explain your answers to the questions above. You may also include additional comments for the author, including concerns about dual publication, research ethics, or publication ethics. (Please upload your review as an attachment if it exceeds 20,000 characters)

Reviewer #1: Clear and concise paper.

I wish the paper included more information on pPEX findings (P, D, C, W), e.g., some background information and if available, proposed etiology. Why were they specifically chosen? Why were they different than Mardin et al.? If possible, please add information and clarify.

🡪 Thank you for your comments. I added the proposed etiology of each sign. The differences between our study and Mardin et al. had been described in discussion part.

Inclusion/exclusion criteria and methods are well-described.

Minor suggestion and correction:
-Simplify the opening sentence (lines 62-65). Stylistically, it is a bit convoluted and not easy to follow.

🡪 Thank you for your comment. Please see the correction in L62-64.
-Correct to show* (line 186).

🡪 Thank you for your comment. I made the correction as per your suggestion.

Reviewer #2: Overall, this is an interesting subject and the paper is well-written. The study design is well done, and the introduction provides a thorough background for the study. The pictures provides very helpful additional information and context for the study.

The authors should clarify why only a portion of the samples were sent for TEM analysis (34 samples), and how those specific samples were chosen.

🡪 Some of the specimens had been lost and fallen dry during transportation to the lab.

The authors should also clarify if any differences were found between the results of the two thicknesses and staining methods (thin samples with toluidine blue, and ultrathin samples with uranyl acetate and lead citrate) used for the samples undergoing TEM analysis.
🡪 The two methods have strong correlation. If ultrathin sectioning missed the lesion that had been observed by thin sectioning, the procedure was repeated.
It would be helpful if the authors could elaborate on the clinical significance of their findings, that pPEX is not associated with PXM and is significantly associated with TEX.

🡪 Thank you for your comment. I added the clinical significance of our findings in L279-283.

Reviewer #3: The study evaluated the ultrastructural alterations of the anterior capsules in patients with signs of preclinical pseudoexfoliation syndrome using histological analyses with light microscopy and transmission electron microscopy in 34 pPEX patients and 62 controls undergoing cataract surgeries in Thailand. The manuscript is well-written with clear logics. The presented data was self-explaining and easy to follow. The authors explained the possible reasons leading to the different outcomes of this study compared to several published reports. The findings here could contribute to the diverse clinical studies in identifying the subclinical features related to PEX diagnosis. The study could be strengthened in a few ways.

1. The introduction justified the importance to diagnose PEX in its early stage. It will be more helpful to add more information about the prevalence of PEX worldwide and the percentage of PEX patients developing elevated IOP and glaucoma eventually. This could help readers understand the different stages of the disease - PEX.

🡪Thank you for your comments. Please see the correction in L71-76. I prefer not to mention the reported prevalence of PEX since it varies significantly due to lack of uniformly conducted epidemiologic studies.
2. TEX diagnosis has been a major finding in this study. However, its diagnosis criteria needs to be detailed in the methods section. Is it necessary to remove any patients with clear clinical signs of TEX in the first place of study enrollment?

🡪 Thank you for your comment. I totally agree with your concern. Eyes with preoperative finding of TEX should be excluded. However, the purpose of this study is to explore the preclinical sign of PEX. We didn’t expect the association of these signs with TEX in the first place. In addition, early diagnosis of TEX needs fully dilated eye examination to see anterior zonular disinsertion. Most (74%) of the eyes assessed revealed incomplete information regarding the TEX stage because of poorly dilated pupil.
3. It has been more than two years after the initial study. Is it possible to check whether any of the study patients has developed clinical symptoms of PEX, especially the one case with TEM alterations?

🡪 Thank you for the comment. I checked each patient on the last follow. Since patients were seen postoperatively by several doctors, PEX material were not carefully evaluated and specifically mentioned.

6. PLOS authors have the option to publish the peer review history of their article ([what does this mean?](https://journals.plos.org/plosone/s/editorial-and-peer-review-process#loc-peer-review-history)). If published, this will include your full peer review and any attached files.


If you choose “no”, your identity will remain anonymous but your review may still be made public.


**Do you want your identity to be public for this peer review?** For information about this choice, including consent withdrawal, please see our [Privacy Policy](https://www.plos.org/privacy-policy).

Reviewer #1: No

Reviewer #2: No

Reviewer #3: No

[NOTE: If reviewer comments were submitted as an attachment file, they will be attached to this email and accessible via the submission site. Please log into your account, locate the manuscript record, and check for the action link "View Attachments". If this link does not appear, there are no attachment files.]

While revising your submission, please upload your figure files to the Preflight Analysis and Conversion Engine (PACE) digital diagnostic tool, <https://pacev2.apexcovantage.com/>. PACE helps ensure that figures meet PLOS requirements. To use PACE, you must first register as a user. Registration is free. Then, login and navigate to the UPLOAD tab, where you will find detailed instructions on how to use the tool. If you encounter any issues or have any questions when using PACE, please email PLOS at [figures@plos.org](mailto:figures@plos.org). Please note that Supporting Information files do not need this step.
